# Detection of somatostatin receptors in human osteosarcoma

**DOI:** 10.1186/1477-7819-6-99

**Published:** 2008-09-10

**Authors:** Markos Ioannou, Panayiotis J Papagelopoulos, Ioannis Papanastassiou, Ioanna Iakovidou, Stamatios Kottakis, Nikolaos Demertzis

**Affiliations:** 1 Department of Orthopaedic Surgery, Cancer Hospital, Pireus, Greece; 2 1st Department of Orthopaedic Surgery, Medical School, University of Athens, Greece; 3 Department of Pathology, Cancer Hospital, Pireus, Greece

## Abstract

**Background:**

The location of osteosarcoma in the metaphysis as well as the age of the patients during the most rapid tumour growth suggest that factors related to skeletal growth are involved in the pathogenesis of this tumour. In this aspect this study aims to detect somatostatin receptors in human osteosarcomas and correlate this finding with the clinical outcome of the tumour.

**Patients and methods:**

Immunohistochemical staining for the presence of somatostatin receptors as well as overall survival and disease free survival rates were retrospectively studied in twenty-nine osteosarcoma patients.

**Results:**

Four osteosarcomas with several aggressive biologic behaviour expressed somatostatin receptors. In these four young patients the event free rate was 0% and the overall survival rate was 50% at 4, 3 years. In contrast the event free survival rate of the twenty-five patients with negative somatostatin receptor status was 72% with an overall survival rate of 76% at 4,3 years.

**Conclusion:**

The present study demonstrates the existence of somatostatin receptors in human osteosarcoma. Tumours expressing somatostatin receptors seemed to be aggressive with a very low disease free and overall survival rate compared to osteosarcoma with negative receptor status.

## Background

Osteosarcoma is the most common primary malignant tumour of bone, with the exception of multiple myeloma. It represents approximately 15% of all biopsy-analyzed primary bone tumours [1,2]. It is most common in males and occurs primarily in the second decade of life. The most common location sites are the metaphysis of bone [3,4]. The age of the patients, coinciding with the adolescent growth spurt as well as the location of tumour sites has led to the syllogism that factors related to skeletal growth are involved in the pathogenesis of this tumour [5-7]. Previous studies maintain that treatment with growth hormone and somatostatin affects the growth of osteosarcoma in animal models [8-10]. Somatostatin is believed to exert antiproliferative effects on tumour cells through receptor-mediated stimulation of tyrosine phosphatase and inhibition of other endogenous growth factors, like growth hormone and insuline-like growth factor 1 [11,12]. In this respect, the presence of somatostatin receptors in human osteosarcoma may have a diagnostic, prognostic and therapeutic value [13].

In this study we aim to detect somatostatin receptors in human osteosarcomas and correlate this finding with the clinical outcome of the tumour.

## Patients and methods

Twenty-nine patients with primary osteosarcoma who were treated at the authors' institution between 1997 and 2006 were included in this study. Fourteen patients were female and fifteen were male. The average age at the time of diagnosis was 27.03 years (range 16–49 years) (Table 1). Preoperative evaluation included precision imaging techniques (plain radiographs, computed tomography and MRI of the lesion, computed tomography of the chest and full body scan with Tc99m). Distribution of anatomic tumour sites was as described in Table 1. The therapeutic protocol included neoadjuvant chemotherapy in all patients with high-dose methotrexate [14-16]. During preoperative chemotherapy one patient died, while we operated on twenty-eight patients aiming at wide resection margins.

**Table 1:** Sex, Age, Location, Surgical Treatment, Outcome and GH receptor status of 29 patients with osteosarcoma.

	**Sex**	**Age**	**Location (Site)**	**Surgical Treatment**	**Oncologic outcome**	**GH receptor status**
1	M	28	Thoracic Spine	LSS	DOD (Died On Disease)	
2	F	16	Distal femur	LSS	NED (No Evident Disease)	
3	F	19	Proximal Tibia	Amputation	DOD	+
4	F	17	Proximal Tibia	Amputation	DOD	+
5	F	39	Distal Femur	LSS	DOD	
6	M	28	Distal Femur	Amputation	NED	
7	M	16	Distal Fibula	LSS	NED	
8	M	22	Distal Femur	LSS	DOD	
9	F	18	Distal Femur	LSS	DOD	
10	F	27	Distal Femur	LSS	NED	
11	F	18	Distal Femur	Amputation	NED	
12	F	35	Proximal Tibia	LSS	NED	
13	M	16	Distal Femur	LSS	NED	
14	M	24	Proximal Humerus	LSS	Disease Progression (Pulmonary metastases)	+
15	M	18	Proximal Tibia	LSS	NED	
16	M	32	Distal Tibia	LSS	NED	
17	F	34	Hip	Died during chemotherapy	DOD	
18	F	49	Proximal Tibia	LSS	NED	
19	F	24	Distal Femur	LSS	NED	
20	M	44	Proximal Humerus	LSS	DOD	
21	F	39	Distal Femur	LSS	Disease Progression (Local recurrence)	
22	F	25	Distal Femur	LSS	NED	
23	M	18	Distal Femur	LSS	NED	
24	M	44	Distal Femur	LSS	NED	
25	M	40	Distal Femur	LSS	NED	
26	M	20	Proximal Humerus	LSS	NED	
27	M	30	Proximal Humerus	LSS	NED	
28	M	16	Proximal Tibia	LSS	Disease Progression (Pulmonary metastases)	+
29	F	28	Proximal Tibia	LSS	NED	

Twenty-four patients underwent a limb salvage procedure, while in four patients amputation was the only surgical option in order to achieve adequate local control.

Disease-free and overall survival was recorded in all patients (table 2).

**Table 2:** Disease free and overall survival rate at 4, 48 years, in 29 patients with osteosarcoma.

	Frequency	Percent
NED (No Evident Disease)	18	62,0
Disease progression	3	10,4
DOD (Died On Disease)	8	27,6
Total	29	100,0

Histological specimens were available for all patients and were reviewed by one experienced pathologist (I.I.). The resected specimens were sliced coronally or axially or both to represent the largest portion of the tumour. The slices were fixed in 10% neutral buffered formaldehyde solution and embedded separately in paraffin. The sections were stained with haematoxylin and eosin and were used for immunohistochemistry. Polyclonal Rabbit Anti-Human somatostatin was used (Dako, Denmark) [17-19] in order to detect the presence of somatostatin receptors [20,21]. The study was approved by the Metaxa Anticancer Hospital Ethical & Scientific Committee.

## Results

**Figure 1:**
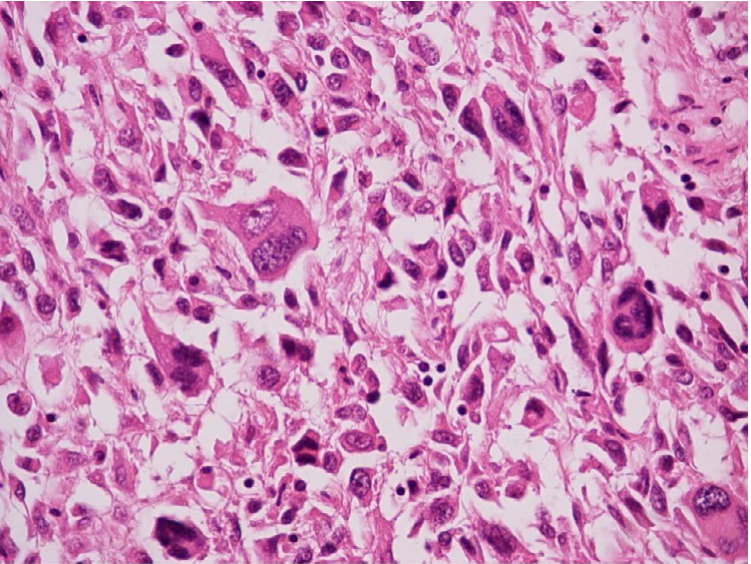
**Osteosarcoma somatostatin negative. Magnification ×400**. This case of an osteosarcoma had no somatostatin receptors. Immunohistochemistry staining with somatostatin did not produce any reaction.

**Figure 2:**
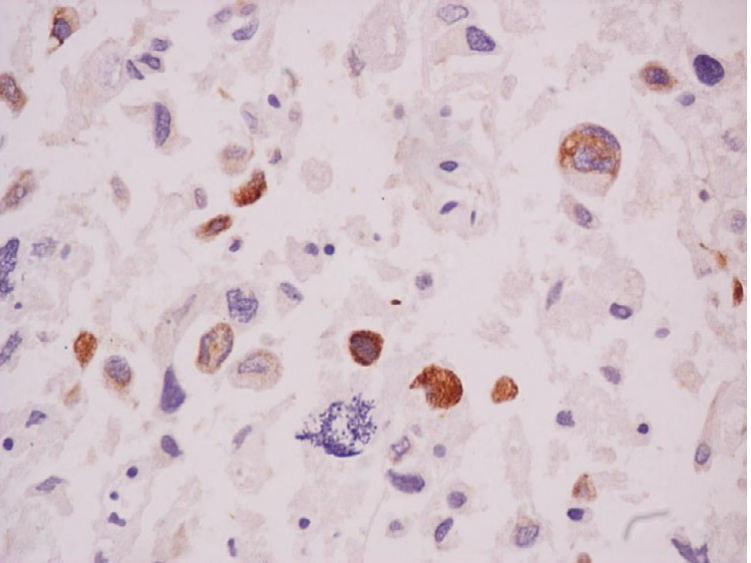
**Osteosarcoma somatostatin positive. Magnification ×630**. In this case staining with somatostatin produced a reaction appearing with an orange zone around the nuclei. This case of osteosarcoma is expressing somatostatin receptors.

Somatostatin receptors were expressed in four osteosarcoma's that exhibited aggressive features (figure 1 and 2). These four tumours appeared in young patients (table 3) with an aggressive biologic behaviour having an event-free rate of 0% and an overall survival rate of 50% at 4.3 years (table 4). In contrast, the event-free survival rate of the twenty-five patients with negative growth hormone receptor status was 72% with an overall survival rate of 76% at 4.3 years.

**Table 3:** Mean Age of patients with positive staining vs. patients with negative staining for receptors of Growth Hormone.

	Patients with Positive staining for receptors of Growth Hormone	Patients with Negative staining for receptors of Growth Hormone
AGE (MEAN/RANGE)	19/16–24 years	28,32/16–49 years

**Table 4:** Disease free and overall survival rate at 4, 48 years in patients with positive staining vs. patients with negative staining for receptors of Growth Hormone.

	Patients with Positive staining for receptors of Growth Hormone (Frequency/Percent)	Patients with Negative staining for receptors of Growth Hormone (Frequency/Percent)
NED (No Evident Disease)	0/0,0	18/72,0
Disease progression	2/50,0	1/4,0
DOD (Died On Disease)	2/50,0	6/24,0
Total	4/100,0	25/100,0

Case one represents a woman, 19-years-old, with a right proximal tibia tumour, stage II B+ on Enneking's staging system [22]. She underwent neoadjuvant chemotherapy followed by femoral amputation. Histological examination revealed grade II osteosarcoma with osteoblastic, as well as chondroblastic areas and 80% tumour necrosis. Two years later there was a local recurrence in the stump of the sciatic nerve, which was treated with hip disarticulation and chemotherapy. Four years post-operative, this patient presented lung metastases, was treated with chemotherapy and eventually died after 1 year. In our retrospective histological study somatostatin receptors were detected.

## Discussion

The use of neoadjuvant chemotherapy in the treatment protocol of osteosarcoma in the late 70's improved disease-free survival, giving a cure rate of 60%–70% for patients with nonmetastatic osteosarcoma of the extremities at presentation [23-25].

Little is known about the aetiology and pathogenesis of this tumour. Genetic predisposition, viral aetiology, irradiation and alkylating agents have been suggested in the pathogenesis of osteosarcoma [3,26,27]. Nowadays, molecular biology seems to be the next step in understanding pathogenesis and improving survival of osteosarcoma. Tumour location in the metaphysis as well as the age of the patients coinciding with the period of rapid body growth suggest that factors related to skeletal growth are involved in the pathogenesis of this tumour.

Somatostatin is characterized as a hormone which inhibits the release of growth hormone from the anterior pituitary gland [28]. The present study demonstrates the existence of somatostatin receptors in human osteosarcoma. Further research is necessary to demonstrate the importance of this finding and its clinical relevance, since there is also evidence from animal studies that treatment with growth hormone and somatostatin affects the growth of osteosarcoma in animal models [8-10]. There is also one study in pediatric patients having metastatic osteosarcoma treated with somatostatin analogue (OncoLar) which shows that the levels of Insulin-like growth factor-1 were reduced. However, this study did not yield significant clinical results [29].

To our knowledge, there is only one study on humans in the literature with 18 osteosarcoma patients where the authors investigated somatostatin receptors by virtue of scintigraphy. In this study a very high incidence of patients with somatostatin receptors was found (up to 75%). The authors found higher incidence in non-metastatic patients and concluded that there is a possible relation between the somatostatin receptors presence and the biological behaviour of the tumour. [30]

A limitation to our study is the small number of specimens that were analyzed, which makes statistical analysis unfeasible; however, because of the novelty of our study and since the tumours expressing somatostatin receptors had a more deleterious course with a very low disease-free and overall survival rate compared to osteosarcoma with negative receptor status, even though the percentage (14%) was much lower than that in the Rizzoli study [30], we believe that this finding should be thoroughly evaluated and investigated with further studies.

## Conclusion

In this study we detected somatostatin receptors in human osteosarcomas. This finding seems to have a prognostic value, predicating a severe aggressive biologic behaviour of the tumour as well as possible therapeutic implications.

## Competing interests

The authors declare that they have no competing interests.

## Authors' contributions

MI drafted the manuscript and carried out the design of the study and performed. II carried out the immunohistochemical studies. PJP, IP and SK participated in the design and coordination of the study and helped to draft the manuscript. All authors read and approved the final manuscript.
